# Breast Cancer Heterogeneity in Latin America: A Scoping Review of Clinical‐Pathological Characteristics, Molecular Subtypes, and Survival

**DOI:** 10.1002/wjs.70096

**Published:** 2025-09-29

**Authors:** María Eugenia Aponte‐Rueda, Fela Mar Gómez‐González, Belén Merck

**Affiliations:** ^1^ Venezuelan Breast Cancer Research and Education Foundation Maracaibo Venezuela; ^2^ Education Department Venezuelan Breast Cancer Research and Education Foundation Caracas Venezuela; ^3^ Department of Medicine & Surgery Faculty of Health Sciences Cardenal Herrera‐CEU University CEU Universities Valencia Spain

**Keywords:** breast cancer, clinical‐pathological characteristics, healthcare disparities, Latin America, molecular subtype, scoping review, survival

## Abstract

**Background:**

Breast cancer in Latin America (LATAM) exhibits distinct clinical‐pathological and molecular features, shaped by genetic diversity and healthcare disparities. This scoping review evaluates these characteristics, focusing on histopathological, molecular subtype, and survival patterns and their implications for future research and public health initiatives.

**Methods:**

A systematic search across MEDLINE (via PubMed), LILACS (Latin American and Caribbean Health Sciences Literature), SciELO (Scientific Electronic Library Online), and Web of Science identified 54 studies across 19 Latin American countries. Data were extracted on histological grading, molecular subtypes, staging, and survival outcomes. Findings were analyzed in the context of regional and global trends.

**Results:**

Fifty‐four studies involving 49,223 women from 19 countries were analyzed. The mean age at diagnosis was 54.3 years. Invasive ductal carcinoma was the most common (79.2%). Advanced‐stage disease (Stages III/IV) was identified in 36.1% of cases. Luminal subtypes were most prevalent (Luminal A: 36.95% and Luminal B: 28.72%), whereas triple‐negative (TNBC) and HER2‐enriched subtypes accounted for 17.45% and 12.69%, respectively. Subtype prevalence varied by country, age, and tumor grade. Five‐year survival rates ranged from 50.5% to 92.5%, with worse outcomes linked to advanced stage, high grade, and TNBC or HER2‐enriched tumors.

**Conclusion:**

Breast cancer in LATAM is characterized by significant heterogeneity in biological subtypes and clinical presentation, often diagnosed at advanced stages, with limited capacity for molecular testing. These findings highlight the urgent need for standardized diagnostic protocols, equitable access to treatment, and region‐specific cancer control strategies to improve outcomes for Latin American women.

## Graphical abstract

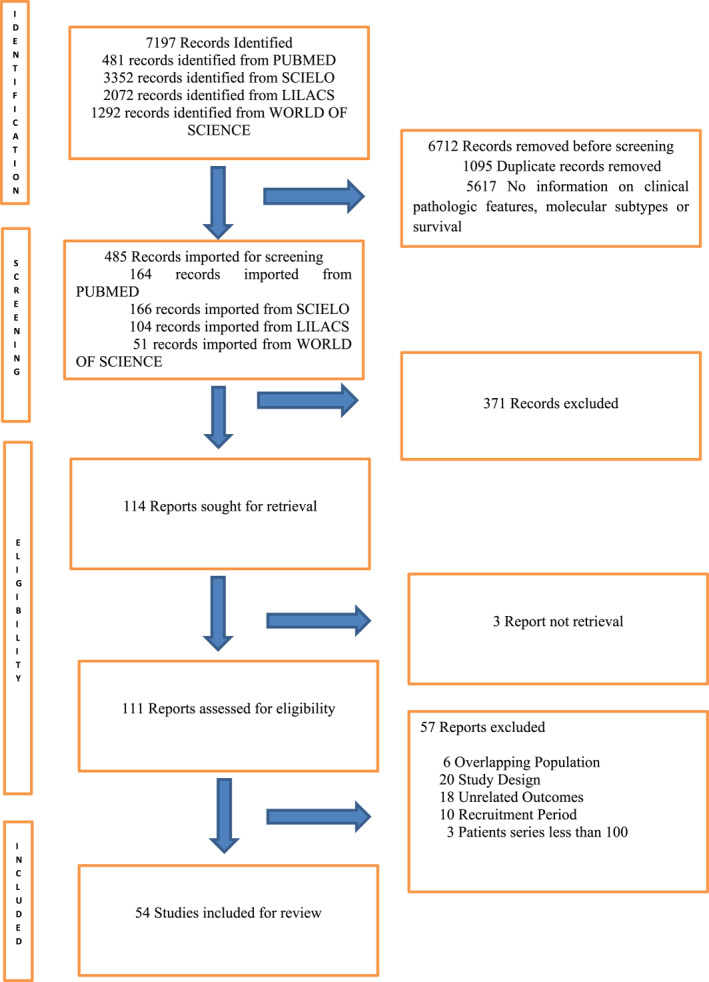

## 1 Introduction

Breast cancer (BC) is the most common malignancy disease and a leading cause of cancer mortality among women worldwide, representing a significant public health challenge. In Latin America (LATAM), BC remains the primary cancer in terms of incidence and mortality among women, accounting for 28.1% of new cases and 16.4% of cancer‐related deaths in the region [1]. Latin America has common cultural, geographical, and historical backgrounds, and its population reflects admixture from Indigenous, African, and European origins [2].

Latin America encompasses 8.3% (approximately 660.3 million people in 2022) of the world's population. This region is experiencing a relatively accelerated demographic transition toward an aging population, with projections indicating that by 2047, individuals aged 60 years and older will outnumber those under 15 years of age [3]. In parallel with this demographic transition, sedentary behavior, obesity, tobacco and alcohol use, and heightened exposure to environmental carcinogens are contributing to the increasing prevalence of noncommunicable diseases [4]. Breast cancer deaths are estimated to increase significantly by 2030, growing from 43,208 to 73,542 cases and further to approximately 94,600 deaths by 2040, with 314,000 new cases each year [5].

Women in Latin America are more likely to experience a higher burden of advanced‐stage disease at diagnosis than their western counterparts [6]. This situation arises from limited access to cancer care and early detection such as mammography and alongside delays in follow‐up after an abnormal mammogram result. Moreover, healthcare systems in Latin America frequently lack universal coverage, with resources unevenly concentrated in select urban centers. Deficiencies in high‐quality health information systems and restricted access to early detection hinder effective breast cancer downstaging and exacerbate disparities in outcomes across the region.

Breast cancer is a heterogeneous disease, encompassing distinct biological entities with varying clinical, histopathological, and molecular characteristics at diagnosis. Prognostic factors, including tumor and patient characteristics, are essential for selecting personalized and effective treatments to improve survival rates and reduce mortality. To address this complexity, localized studies in Latin America serve as an essential prerequisite for establishing equitable care, as significant gaps in data in the region hinder the application of precision medicine.

Currently, the existing data on Latino patient characteristics primarily derive from studies of United States (US) Hispanic cohorts [7], which may not fully reflect the genetic and sociodemographic diversity of the Latin America population. The available data on breast cancer in Latin America are scarce, fragmented, and lack regional representativeness, which hampers accurate characterization of the population and reinforces the persistent underrepresentation of these patients in global cancer research, despite their marked genetic and sociocultural diversity. By emphasizing the clinical and molecular heterogeneity of BC in the region, this scoping review aims to consolidate, summarize, and critically appraise the existing literature to address key knowledge gaps in this field. Specifically, we sought to answer the following questions:

1. What are the primary features of breast cancer, and how do they differ across the region?
2. What methodologies have been used to study these features, and what gaps in knowledge still exist?

## 2 Methods

A scoping review was conducted in accordance with the recommendations of the PRISMA‐ScR checklist for operational steps (Supporting Information S1). The study protocol are available at https://osf.io/gfsxa/files/osfstorage on the Open Science Framework website. The literature search was conducted across MEDLINE via PubMed, SciELO (Scientific Electronic Library Online), LILACS (Latin American and Caribbean Health Sciences Literature), and Web of Science, focusing on articles published through July 1, 2024, and limited to those published after 2009. Articles written in English, Portuguese, or Spanish were included, whereas gray literature was excluded to focus on peer‐reviewed publications. The search strategy employed Medical Subject Headings (MeSH) terms, including the term “breast cancer” and its synonyms, as well as the names of the countries, along with their corresponding demonyms (Table 1). Manual searching of the reference lists of included studies ensured that additional relevant publications were identified.

**TABLE 1:** Search terms.

PubMed (“Latin America” [MESH] OR “Central America” [tw] OR “South America” [tw] OR Argentina [tw] OR Argentinean [tw] OR Brazil [tw] OR Brazilian [tw] OR Bolivia [tw] OR Bolivian [tw] OR Chile [tw] OR Chilean [tw] OR Colombia [tw] OR Colombian [tw] OR “Costa Rica” [tw] OR “Costa Rican” [tw] OR Cuba [tw] OR Cuban [tw] OR “Dominican Republic” [tw] OR Dominican [tw] OR Ecuador [tw] OR Ecuadorian [tw] OR “El Salvador” [tw] OR Salvadoran [tw] OR Guatemala [tw] OR Guatemalan [tw] OR Haiti [tw] OR Haitian [tw] OR Honduras [tw] OR Honduran [tw] OR Mexico [tw] OR Mexican [tw] OR Nicaragua [tw] OR Nicaraguan [tw] OR Panama [tw] OR Panamanian [tw] OR Paraguay [tw] OR Paraguayan [tw] OR Peru [tw] OR Peruvian [tw] OR Uruguay [tw] OR Uruguayan [tw] OR Venezuela [tw] OR Venezuelan [tw]) AND (“Breast Neoplasms” [MeSH] OR “breast cancer” OR “breast carcinoma” OR “breast tumors” OR “cancer of breast”) AND (“Observational Studies as Topic” [MeSH] OR “observational study” OR “cross‐sectional study” OR “cohort study” OR “case series”)
Scientific Electronic Library Online: SciELO (Latin America OR Central America OR South America OR Argentina OR Brazil OR Bolivia OR Chile OR Colombia OR Costa Rica OR Cuba OR Dominican Republic OR Ecuador OR El Salvador OR Guatemala OR Haiti OR Honduras OR Mexico OR Nicaragua OR Panama OR Paraguay OR Peru OR Uruguay OR Venezuela) AND (breast cancer) AND (observational studies)
Latin American and Caribbean Health Sciences Literature: LILACS (Latin America OR Central America OR South America OR Argentina OR Brazil OR Bolivia OR Chile OR Colombia OR Costa Rica OR Cuba OR Dominican Republic OR Ecuador OR El Salvador OR Guatemala OR Haiti OR Honduras OR Mexico OR Nicaragua OR Panama OR Paraguay OR Peru OR Uruguay OR Venezuela) AND (breast cancer) AND (observational studies)
Web of Science (“Latin America” OR “Central America” OR “South America” OR Argentina OR Argentinean OR Brazil OR Brazilian OR Bolivia OR Bolivian OR Chile OR Chilean OR Colombia OR Colombian OR “Costa Rica” OR “Costa Rican” OR Cuba OR Cuban OR “Dominican Republic” OR Dominican OR Ecuador OR Ecuadorian OR “El Salvador” OR Salvadoran OR “Guatemala OR Guatemalan OR Haiti OR Haitian OR Honduras OR Honduran OR Mexico OR Mexican OR Nicaragua OR Nicaraguan OR Panama OR Panamanian OR Paraguay OR Paraguayan OR Peru OR Peruvian OR Uruguay OR Uruguayan OR Venezuela OR Venezuelan) AND (“Breast Neoplasms” OR “breast cancer” OR “breast carcinoma” OR “breast tumors” OR “cancer of breast”) AND (“Observational Studies as Topic” OR “observational study” OR “cross‐sectional study” OR “cohort study” OR “case series”)

Two reviewers (M.E.A.‐R. and B.M.) independently screened the titles and abstracts of all records to assess relevance and identify studies that fulfilled the inclusion criteria, specifically studies focusing on women with confirmed BC diagnoses living in Latin America. The exclusion criteria were as follows: (1) studies that concentrated solely on specific disease stages, age groups, histological types, or molecular subtypes; (2) studies with fewer than 100 patients; (3) studies involving individuals from the same institution to prevent duplication of patients; and (4) multi‐country studies that failed to report results by individual country.

Data were extracted in duplicates and independently by the review authors. Disagreements were resolved through consensus. The database spreadsheet was built in Microsoft Excel (Microsoft Corporation, Redmond, WA, USA), and was designed to extract the following information: general study data (study design, years of diagnosis, number of included patients, mean age, and study setting), tumor characteristics (TNM staging, histological type, tumor grade, molecular subtype, hormonal receptor, and HER‐2 status), and overall survival. For studies reporting immunohistochemistry (IHC) and molecular subtype data, the following information was retrieved: the IHC method, covariates, and surrogates' intrinsic subtypes. For studies reporting survival data, we compiled information regarding facility type, survival analysis methods, overall survival, disease‐specific survival (or both), covariates, and significant variables influencing survival outcomes. This scoping review was designed to provide an overview of the existing evidence base regardless of methodological quality. Therefore, a formal assessment of the methodological quality of the included studies was not performed [8].

## 3 Results

**FIGURE 1:**
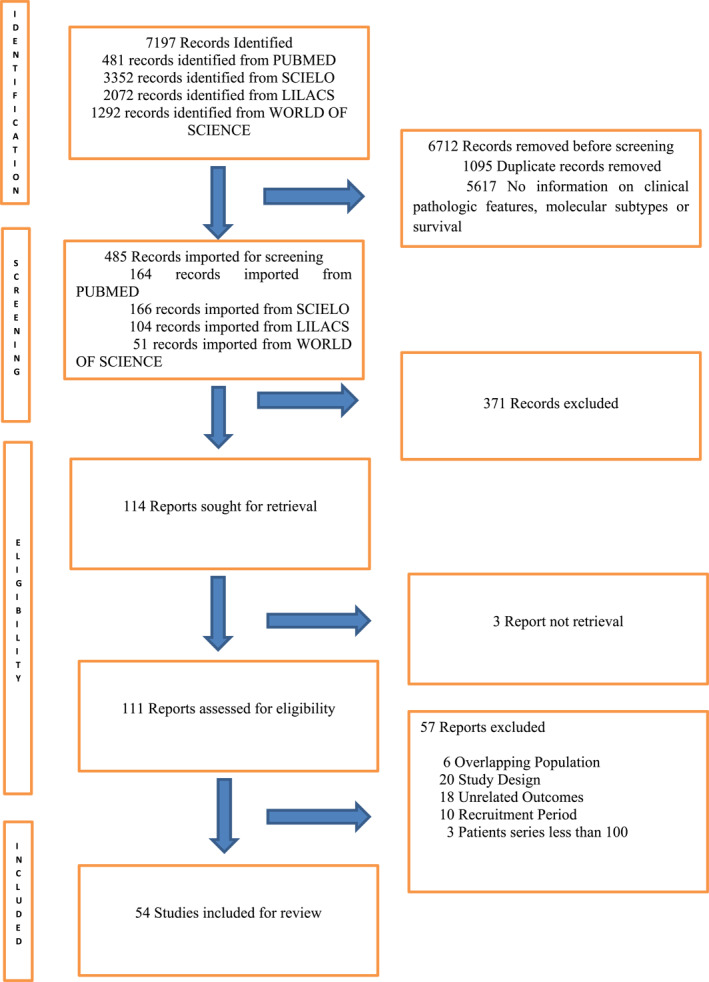
Study selection flowchart.

The electronic database search identified 485 titles. Of these, 371 were excluded because they did not address the research questions. After screening titles and abstracts, 114 studies were selected for full‐text review. Ultimately, 54 articles [9, 10, 11, 12, 13, 14, 15, 16, 17, 18, 19, 20, 21, 22, 23, 24, 25, 26, 27, 28, 29, 30, 31, 32, 33, 34, 35, 36, 37, 38, 39, 40, 41, 42, 43, 44, 45, 46, 47, 48, 49, 50, 51, 52, 53, 54, 55, 56, 57, 58, 59, 60, 61, 62] were included in the final analysis (Figure 1), whereas 60 were excluded due to failure to meet the inclusion criteria.

The majority of studies were conducted in Brazil (*n* = 10) [9, 10, 11, 12, 13, 14, 15, 16, 17, 18] and Mexico (*n* = 8) [19, 20, 21, 22, 23, 24, 25, 26], followed by Peru (*n* = 5) [27, 28, 29, 30, 31], Colombia (*n* = 5) [32, 33, 34, 35, 36], Venezuela (*n* = 4) [37, 38, 39, 40], Cuba (*n* = 4) [41, 42, 43, 44], Argentina (*n* = 3) [45, 46, 47], and Ecuador (*n* = 3) [48, 49, 50]. Additional countries with fewer than three studies included Haiti (*n* = 2) [51, 52], Bolivia (*n* = 1) [53], Chile (*n* = 1) [54], Costa Rica (*n* = 1) [55], El Salvador (*n* = 1) [56], Guatemala (*n* = 1) [57], Honduras (*n* = 1) [58], Panama (*n* = 1) [59], Paraguay (*n* = 1) [60], Puerto Rico (*n* = 1) [61], and Uruguay (*n* = 1) [62]. Puerto Rico was included in this study due to its geographical location and cultural background, despite being an unincorporated territory of the US. Collectively, these studies encompassed 49,223 native women diagnosed with BC between 2009 and 2023.

Three studies [53, 58, 60] did not strictly meet the inclusion criteria but were retained for analysis: one study from Honduras [58] was included despite lapses in the recruitment period. Studies from Bolivia [53] and Paraguay [60], each with fewer than 100 patients, were included as they represent the sole available data from these countries.

Most studies were conducted in single‐institution settings (*n* = 43) [9, 11, 12, 13, 14, 15, 16, 18, 19, 20, 21, 22, 23, 24, 25, 26, 27, 28, 29, 30, 31, 32, 34, 36, 37, 38, 39, 40, 41, 42, 43, 44, 48, 49, 50, 51, 53, 54, 55, 56, 57, 58, 60], whereas a smaller number were multicentric (*n* = 11) [10, 17, 33, 35, 45, 46, 47, 52, 59, 61, 62].

The relevant findings were organized into three key areas: (1) clinical‐pathological characteristics (*n* = 53) [9, 10, 11, 12, 13, 14, 15, 16, 17, 18, 19, 20, 21, 22, 23, 24, 25, 26, 27, 28, 29, 30, 31, 32, 33, 34, 35, 36, 37, 38, 39, 41, 42, 43, 44, 45, 46, 47, 48, 49, 50, 51, 52, 53, 54, 55, 56, 57, 58, 59, 60, 61, 62], (2) tumor receptor expression and surrogate molecular subtypes (*n* = 35) [10, 15, 17, 18, 20, 21, 22, 23, 24, 25, 26, 27, 28, 29, 30, 31, 33, 35, 37, 38, 39, 40, 43, 45, 46, 48, 49, 50, 52, 53, 55, 57, 60, 61], and (3) overall survival (*n* = 28) [10, 11, 12, 13, 14, 15, 16, 17, 18, 19, 21, 23, 25, 26, 27, 28, 32, 34, 38, 39, 41, 42, 45, 51, 52, 53, 55, 61].

### 3.1 Clinical‐Pathological Characteristics

A total of 53 studies [9, 10, 11, 12, 13, 14, 15, 16, 17, 18, 19, 20, 21, 22, 23, 24, 25, 26, 27, 28, 29, 30, 31, 32, 33, 34, 35, 36, 37, 38, 39, 41, 42, 43, 44, 45, 46, 47, 48, 49, 50, 51, 52, 53, 54, 55, 56, 57, 58, 59, 60, 61, 62] from 19 countries were analyzed (Table 2). The mean age reported in 43 studies was 54.28 ± 2.75 years, with a range of 49–60.9 years, consistent with findings from an intercontinental report [63] in which the mean age in Asia and Latin America (57 ± 13 years) was significantly lower than in Europe (61 ± 13 years). The most frequent histological subtype was invasive ductal carcinoma, with a mean prevalence of 79.25% ± 18.65%, consistent with reports from other studies reporting approximately 75% of cases [64]. Invasive lobular carcinoma was the second most common subtype, with a mean frequency of 7.80% ± 4.09%, aligning with international data reporting rates ranging from 9% in Asia to 15% in Europe [63].

**TABLE 2:** Clinicopathologic features of selected studies of patients with breast cancer in Latin America.

Author/Country (year)	Study design	Patients	Years of diagnosis	Study setting	Mean age	Stage at presentation (%)	Histological grading (%)	Histological type (%)
Abriata et al. [45]/Argentina (2019)	Case series	4883	2012–2016	Institutional Cancer Registry of Argentina (RITA)	57.6	ST1: 8.5, ST2: 18.6, ST3: 6.4, ST4: 6.4, and STUKN: 49.3	G1: 8.6, G2: 40.2, and G3: 20.7	IDC: 76 ILC: 8
González Cortez et al. [46]/Argentina (2020)	Case series	5226	2006–2016	Breast Cancer Registry (RCM)	56	ST1: 76.5 and ST2: 13.5; ST3: 10.0	NR	IDC: 72.8 ILC: 11.3
Meiss et al. [47]/Argentina (2012)	Cohort	607	2012–2013	The Collaborative Group for the Study of Female Breast Cancer	57.5	ST1: 37.7, ST2: 37.5, ST3: 18.8, and ST4: 1.5 STUKN: 16.6	G1: 14, G2: 43, and G3: 28.8 GUKN: 13.9	IDC: 87.0 ILC: 13.0
Cruz‐Guisbert [53]/Bolivia (2019)	Cohort	69	2000–2016	Social Security University Hospital	51	ST2: 26.6 and ST4: 32.8	NR	NR
Reis et al. [9]/Brazil (2020)	Cross‐sectional	137	2015–2018	High Complexity Care Unit in Oncology (UNACON) of the city of Imperatriz, state of Maranhão	52.1	≤ STIIB: 47.4 > STIIB: 52.6	NR	IDC: 84.7 ILC: 10.2
Peres et al. [10]/Brazil (2023)	Cohort	1654	2000–2018	São Paulo's hospital‐based cancer registry (RHC/SP) and the Immunohistochemistry Laboratory database at the Oncocenter Foundation of São Paulo (FOSP)	56.8	ST1: 17.4 and ST2: 45.8 ST3: 29.6 ST4: 7.2	NR	IDC: 71.9 ILC: 3.6
Fujimoto et al. [11]/Brazil (2019)	Cohort	161	2007–2012	High Complexity Care Unit in Oncology (UNACON) of the state of Acre, Rio Branco, western Amazon	52.8	NR	NR	NR
Ayala et al. [12]/Brazil (2019)	Cohort	471	2000–2014	Unified Health System Mastology Service in Joinville, State of Santa Catarina	55.3	ST1: 13.4 and ST2: 40.8 ST3: 38.3 and ST4: 7.5	G1: 9.1, G2: 54.5, and G3: 36.4	NR
Balabram et al. [13]/Brazil (2013)	Cohort	897	2001–2008	Clinical Hospital of the Federal University of Minas Gerais	55.32	ST1: 24.86 and ST2: 35.12 ST3: 40.02	G1: 20.18 G2: 42.92 G3: 35.67	IDC: 84.73 ILC: 8.8
Schneider et al. [14]/Brazil (2009)	Cohort	1008	2000–2002	Santa Catarina Center for Cancer Research and the Irmandade Nosso Senhor dos Passos Charity Hospital, Florianópolis	53.85	ST1: 18.1; ST2: 46.2; ST3: 24.3; ST4: 11.4	NR	IDC: 77.7
de Macêdo Andrade et al. [15]/Brazil (2014)	Cohort	269	2013	“Fundação de Assistência da Paraíba” (FAP) public hospital of Campina Grande, Paraíba, Brazil	55.36	NR	NR	NR
Fayer et al. [16]/Brazil (2016)	Cohort	195	2000–2001	High Complexity Care Unit in Oncology (UNACON) of the municipality of Juiz de Fora, state of Minas Gerais	57.8	ST1: 23.4 and ST2: 52.6 ST: 20.3 and ST4: 3.6	NR	NR
Simon et al. [17]/Brazil (2019)	Cohort	2296	2008–2009	The AMAZONA study	54	ST1: 23.3 and ST2: 53.5 ST3: 23.2	G1: 10.8, G2: 56.7, and G3: 22.5	IDC: 92 ILC: 6.5
Marques et al. [18]/Brazil (2022)	Cohort	1278	2010–2014	Cancer facility in the state of Sergipe	55	ST1: 15.3 and ST2: 32.2 ST3: 27.2 and STUKN: 22.9	NR	IDC: 90.1 ILC: 4.2
Ceballos‐Morales et al. [54]/Chile (2021)	Cross‐sectional	1077	2005–2015	The breast unit of las Higueras health service in Talcahuano	60	ST1: 20.0 and ST2: 45.6 ST3: 17.7 and ST4: 3.6	NR	IDC: 73.5 ILC: 5.4
Zuluaga‐Liberato et al. [32]/Colombia (2016)	Cohort	228	2005–2013	The oncological center in Bogota	49.61	ST1: 12.3 and ST2: 63.2; ST3: 20.5 and ST4: 2.6	G1: 12.3, G2: 55.3, and G3: 30.3	IDC: 86.8 ILC: 6.6
Serrano‐Gomez et al. [33]/Colombia (2016)	Cohort	301	2008–2012	Colombian National Cancer Institute and Caribbean University Hospital	56.6	ST1: 7.3, ST2: 38.9, ST3: 44.2, and ST4: 2.0 STUKN: 7.6	G1: 5.6, G2: 51.8, and G3: 29.6	IDC: 92.7 ILC: 2.3
Pardo et al. [34]/Colombia (2018)	Cohort	1928	2007, 2010, and 2012	Colombian National Cancer Institute	55	ST1: 5.8, ST2: 28.3, ST3: 39.0, and ST4: 7.3 STUKN: 19.7	NR	NR
Toro‐Castaño et al. [35]/Colombia (2022)	Cross‐sectional	377	2015–2018	Pathology laboratory from the Colombian coffee‐growing region and the Caldense Institute of Pathology, Manizales	51–60	NR	NR	IDC: 89.9; ILC: 6.1
Ramírez‐Martínez et al. [36]/Colombia (2015)	Cross‐sectional	1480	2006–2013	The breast cancer unit in Medellín	54	Early stage: 32 Locally advanced: 48 Metastatic stage: 3	G1: 15 and G2: 39	IDC: 80 ILC: 7
Srur‐Rivero et al. [55]/COSTA RICA (2014)	Cohort	199	2009–2010	San Juan de Dios Hospital	53	ST1: 17.6 ST2: 41.71 and ST3: 33.6 ST4: 3.5	G1: 13.6, G2: 37.2, and G3: 31.1	NR
Ricardo‐Ramírez et al. [41]/Cuba (2013)	Cohort	132	2002–2012	Saturnino Lora Torres Hospital, Santiago de Cuba	NR	NR	NR	NR
García Soto et al. [42]/Cuba (2019)	Cohort	288	2010–2015	José Ramón López Tabrane, Hospital, Matanzas	NR		NR	NR
Ramírez Valle et al. [43]/Cuba (2019)	Cross‐sectional	418	2012–2016	Third Congress Hospital, Pinar del Río	51–60	NR	NR	IDC: 59.1 ILC: 15.3
Torres Ajá [44]/Cuba (2013)	Cross‐sectional	377	2007–2011	Cienfuegos Hospital	NR	ST1: 13.7 and ST2: 62.8; ST3: 17 and ST4: 1.5	NR	IDC: 70.29 ILC: 12.20
González‐Longoria et al. [48]/Ecuador (2022)	Cross‐sectional	199	2014–2017.	Abel Gilbert Pontón Hospital	56.77	NR	G2: 56.8	IDC: 99.5
Vela et al. [49]/Ecuador (2020)	Cross‐sectional	147	2016–2019	Metropolitan Hospital, Quito	60.9	NR	NR	NR
Ulloa et al. [50]/Ecuador (2020)	Cohort	284	2006–2013	National Oncological Institute	40–65	NR	NR	IDC: 83.09 ILC: 5.63
Orellana Beltrán et al. [56]/El Salvador (2021)	Case series	344	2017–2018	National Cancer Institute	54	NR	NR	IDC: 83.4 ILC: 4.9
Salazar‐Cifuentes et al. [57]/Guatemala (2017)	Case series	279	2012–2017	Guatemalan Social Security Institute	NR	NR	NR	NR
Duarte Muñoz et al. [58]/Honduras (2011)	Cross‐sectional	685	1999–2009	Emma Romero de Callejas Cancer Center	46–55	ST1: 12.7, and ST2: 42.4 ST3: 27.1 and ST4: 11	NR	IDC: 78.2 ILC: 15.7
Fadelu et al. [51]/Haiti (2020)	Cohort	341	2012–2016	University Hospital Mirebalais	49	ST1‐ST2: 27.3; ST3‐ST4: 55.1	G1: 22.3; G3: 36.7	IDC: 65.7; ILC: 3.5
DeGennaro et al. [52]/Haiti (2018)	Cohort	525	2013–2017	Hospital Bernard Mevs and St Luke's Hospital in Port‐au‐Prince	49.1	ST1: 0.4 and ST2: 15.2 ST3: 55.5 and STIV: 28.4	G1: 13.3, G2: 48.2, and G3: 34.9	IDC: 87.3
Álvarez‐Bañuelos et al. [19]/Mexico (2016)	Cohort	114	2009	State Cancer Center in Xalapa, Veracruz	41–59	NR	G1: 2.2, G2: 50.0, and G3: 47.7	IDC: 84.2 ILC: 6.14
Maffuz‐Aziz et al. [20]/Mexico 2017	Cohort	4411	2005–2014	Breast Diseases Institute (IEM‐FUCAM)	53.7	STI‐STIIA: 36.4, STIIB‐ST3C: 45, ST4: 7.7, and STUKN: 3.9	G1: 9.1, G2: 54.1, and G3: 34.6	IDC: 79.7 ILC: 7.8
Reynoso‐Noverón et al. [21]/Mexico (2017)	Cohort	4300	2007–2013	National Cancer Institute (INCan), Mexico City	52	ST1: 14.2 and ST2: 36.6 ST3: 36.2 and ST4: 12.9	G1: 18.5, G2: 30.1, and G3: 53.1	IDC: 85.1 ILC: 9.4
Reyna‐Sevilla et al. [22]/Mexico(2021)	Cross‐sectional	1840	2013–2017	Jalisco Cancer Institute (IJC), Guadalajara, Jalisco	53.2	ST1, ST2: 35.5, 3 and ST3ST4: 53.1	G1: 13.2, G2: 60.1, and G3: 26.7	IDC: 88.9 ILC: 8.9
Ornelas‐Aguirre et al. [23]/Mexico (2013)	Cross‐sectional	768	2006–2012	Mexican Social Security Institute, Northwest National Medical Center, Sonora	53.07	ST1 + ST2: 76.2, ST3: 1.7; ST4: 3.5 and STUKN: 18.6	G1: 9.2, G2: 23.4, and G3: 67.3	NR
Pastén‐Zapata et al. [24]/Mexico (2019)	Cross‐sectional	136	2013–2018	Private Hospital in Monterrey	54	ST1–ST2B: 87.5 and ST4: 3.7	NR	IDC: 94.9 ILC2.2
Valle‐Solís et al. [25]/Mexico (2019)	Cohort	303	2004–2015	National Medical Center, November 20th, Social Security Institute, Mexico City	51–56	ST1–2a: 35.64 and STIIb–ST3: 47.85 ST4: 7.2	NR	NR
Heredia‐Caballero et al. [26]/Mexico (2018)	Cohort	119	2010	Women's Military Hospital, Mexico City	56.2	ST1: 15.12 and ST2: 62.96 ST3: 16.8 and ST4: 3.36	G1: 13, G2: 29, and G3: 33	IDC: 76 ILC: 13
López Castillo et al. [59]/Panama (2022)	Cohort	4134	2012–2016	Panama's National Cancer Registry/National Oncological Institute	58	ST1: 15.1, ST2: 39.1, ST3: 33.6, and ST4: 11.2	G2: 35.2	NR
Cabrera et al. [60]/Paraguay (2019)	Cross‐sectional	75	2016–2017	Saint Paul Hospital	52	ST1: 2.70 and ST2: 59.46 ST3: 28.38 and ST4: 9.46	G1: 5.9, G2: 53.7, and G3: 40.4	IDC: 91 ILC: 9
Medina Bueno [27]/Peru (2017)	Cohort	253	2009–2012	Carlos Alberto Seguín Escobedo National Hospital	56	ST1: 23.9; ST2: 46.4; ST3: 29.7	G1: 16.4; G2: 63.4; G3: 20	IDC: 82.1; ILC: 8.6
Garcés et al. [28]/Peru (2012)	Cohort	2047	2000–2005	National Institute of Neoplastic Diseases, Lima	51.2	ST1: 16.7 and ST2: 50.2 ST3: 29.1	G1: 91.11, G2: 6.3, and G3: 0.1	IDC: 91.1 ILC: 6.3
Chachaima‐Mar et al. [29]/Peru (2020)	Cross‐sectional	259	2015–2017	Arzobispo Loayza National Hospital, Lima	54.64	NR	G1: 23.94 and G2: 53.28 G3: 22.78	IDC: 88.03 ILC: 5.41
Abad‐Licham et al. [30]/Peru (2018)	Case series	157	2008–2015	Oncological Institute of the North of Peru	55	NR	G1: 3.2, G2: 48.4, and G3: 48.4	IDC: 91.1 ILC: 7
Galindo‐Céspedes et al. [31]/Peru (2016)	Case series	271	2012–2015	Almanzor Aguinaga Asenjo Hospital, Chiclayo	56.19	NR	NR	IDC: 90.7 ILC: 4.06
Ortiz et al. [61]/Puerto Rico (2013)	Cohort	663	2002–2005	I. González Martínez Oncologic Hospital and the Auxilio Mutuo Hospital	57.0	Localized: 61.60; Regional/distant: 38.40	G1: 15.19 and G2: 46.64 G3: 33.04	IDC: 75.99 ILC: 17.02
Castillo et al. [62]/Uruguay (2012)	Cohort	169	2006–2008	Clinics Hospital, Army Central Hospital, Medical Center of the Medical Trade Union, National Cancer Institute	55	ST1: 31.3 and ST2: 46.1 ST3: 22.4	G1 + G2: 59.7 and G3: 34.9	IDC: 87 ILC: 13
Fernández‐Tortolero et al. [37]/Venezuela (2021)	Cohort	209	2014–2016	Oncological Institute “Dr. Miguel Pérez Carreño,” Valencia	51.6	ST1: 2.9 and ST2: 40.2 ST3: 52.6 and ST4: 4.3	G1: 23.4, G2: 57.9, and G3: 18.7	IDC: 100
Bolívar Abreu et al. [38]/Venezuela (2013)	Cohort	266	2003–2005	Oncological Institute “Dr. Luis Razetti,” Caracas	51.2	ST1: 14.29 and ST2: 49.25 ST3: 31.95 and ST4: 4.51	G1: 24.44 and G2: 54.51 G3: 21.05	IDC: 76.31 ILC: 7.89
Chien et al. [39]/Venezuela (2012)	Cohort	312	2000–2008	Oncological Institute “Dr. Miguel Pérez Carreño,” Valencia	50.84	ST1: 9.29 and ST2: 38.46 ST3: 41.67 and ST4: 10.58	G1: 14.10 and G2: 54.17 G3: 31.73	NR

Abbreviations: G1: Grade I; G2: Grade II; G3: Grade III; IDC: invasive ductal carcinoma; ILC: invasive lobular carcinoma; NR: not reported.; ST1: Stage I; ST2: Stage II; ST3: Stage III; ST4: Stage IV; Stage Unknown: STUKN.

The distribution of cancer stages at diagnosis was reported in 35 studies [10, 12, 13, 14, 16, 17, 18, 20, 21, 22, 23, 25, 26, 27, 28, 32, 33, 34, 36, 37, 38, 39, 44, 45, 46, 47, 51, 52, 54, 55, 58, 59, 60, 61, 62]: Stage I: mean of 19.06% ± 15.71%, Stage II: mean of 40.88% ± 13.63%, Stage III: mean of 28.14% ± 12.06%, and Stage IV: mean of 7.99% ± 7.55%. There is substantial global variation in the stage at which BC is diagnosed. An intercontinental study [63] reports that early‐stage BC (Stages I–II) is most prevalent in the US (49.0% and 34.0%, respectively), followed by Asia (23.4% and 43.9%), Europe (22.0% and 37.7%), and Latin America (18.2% and 35.3%). In Latin America, 26.3% of patients are diagnosed at Stage III, a proportion more than twice observed in the U.S. (11.0%), and significantly higher than those observed in Europe (14.1%) and Asia (18.8%) [63].

Histological grade was reported in 27 studies [12, 13, 17, 18, 19, 20, 21, 22, 23, 26, 27, 28, 29, 30, 32, 33, 36, 37, 38, 39, 45, 47, 51, 52, 55, 60, 61, 62], revealing the following average distribution: Grade I, 13.20% (range: 9.5%–17.76%); Grade II, 48.40% (range: 36.2%–54.34%); and Grade III, 31.10% (range: 21.77%–36.19%). These findings reflect a consistent pattern across studies, with a predominance of intermediate‐ and high‐grade tumors. This trend aligns with data from a large and extensive international review, which reported broader but comparable ranges: Grade I, 17%–38%; Grade II, 36%–49%; and Grade III, 19%–46% [65].

### 3.2 Molecular Subtypes

We identified 32 articles [9, 10, 15, 17, 20, 21, 22, 23, 24, 25, 26, 27, 28, 29, 30, 31, 33, 35, 37, 38, 39, 40, 43, 45, 46, 49, 50, 52, 53, 55, 57, 60, and 61] that reported surrogate molecular classifications, encompassing luminal A/B, triple‐negative breast cancer (TNBC), and HER2‐enriched subtypes (Table 3). Among these, nine studies [9, 10, 15, 27, 29, 30, 33, 35, and 48] classified subtypes according to the St. Gallen Consensus, whereas nine studies [20, 21, 25, 38, 45, 46, 52, 53, and 55] did not detail the classification approach for luminal subtypes. Additionally, eight studies [17, 23, 39, 40, 43, 49, 50, and 57] used alternative nomenclature for subtypes; five studies [18, 24, 26, 48, 60] failed to report surrogate classification, and four studies [22, 28, 31, 61] adopted a simplified nomenclature for luminal subtypes, categorizing them as follows: Luminal A (ER+/PR+, HER2−) and Luminal B (ER+/PR+, HER2+). Thirteen studies [10, 15, 20, 21, 27, 28, 29, 30, 33, 35, 37, 39, and 61] provided percentages for hormone receptor positivity.

**TABLE 3:** Molecular subtype of selected studies with breast cancer in Latin America.

Author/Year	LUM A (%)	LUM B (%)	Triple negative (%)	Her2‐enriched (%)	Other immunohistochemistry profile (%)	Immunohistochemical assessment	Surrogate molecular classification	Study significant variable
Abriata et al. [45]/Argentina (2019)	—	—	5.8	4.2	Luminal: 0 Other: 13.1 Unknown: 36.9	NR	ER+ and PR+, HER2−, ER− and PR−, and HER2+; Triple negative: ER−, PR−, and HER2‐ and triple positive: ER+, PR+, and HER2+	36‐months OS
González Cortez et al. [46]/Argentina(2020)	—	—	9.30	12.60	Luminal: 78.1	NR	NR	Age and stage at diagnosis and histological grading
Cruz‐Guisbert [53]/Bolivia (2019)	—	—	6.30	29.70	HR+: 64.1	NR	NR	
Marques et al. [18]/Brazil (2022)	11.00	30.60	15.70	4.90	LUMINAL X: 27.5	HER2: IHC profiling and FISH test	NR	5 years OS
Reis et al. [9]/Brazil (2020)	19.70	49.60	13.10	12.40	Inconclusive: 5.1	IHC profiling and FISH test	2013 St. Gallen Consensus [66]	Stage at diagnosis, tumor size, lymph node involvement, and metastasis
Peres et al. [10]/Brazil (2023)	29.00	47.50	15.20	8.20	ER+: 74.1 PR+: 66.1 Her2Neu+: 14.5	Samples with at least 1% estrogen‐ or progesterone‐positive tumor nuclei were considered positive HER2: IHQ profiling and FISH test [67]	2011 St. Gallen Consensus [68]	5 years OS and Age at diagnosis
de Macêdo Andrade et al. [15]/Brazil (2014)	23.79	44.61	17.10	14.50	ER: 66.54 PR: 56.51 HER2 Neu: 49.07	ER and PR positivity were defined as any positive nuclear staining in ≥ 1% of tumor cells HER2: IHQ profiling and FISH test	2011 St. Gallen Panel [68]	Age at diagnosis, tumor size, lymph node status, metastasis, and histological grading
Simon et al. [17]/Brazil (2019)	49.40	21.90	2.00	7.70		NR	[69]	Stage at diagnosis
Serrano‐Gomez et al. [33]/Colombia (2016)	26.20	37.20	11.60	8.60	Basal‐like: 9	ER and PR positivity were defined as any positive nuclear staining in ≥ 1% of tumor cells HER2: IHQ profiling and FISH test [70]	2013 St. Gallen Consensus [66]	Age at diagnosis, tumor size, lymph node status, and histological grading
Toro‐Castaño et al. [35]/Colombia (2022)	22.30	56.20	14.90	6.60	ER+ 8.5, ER−: 21.5, PR+: 64.7, PR−: 35.3, and Her2+: 17.5	ER and PR positivity were defined as any positive nuclear staining in ≥ 1% of tumor cells HER2: IHQ profiling and FISH test [71]	2011 St. Gallen Consensus [68]	
Srur‐Rivero et al. [55]/Costa Rica (2014)	—	—	17.10	9.50	HR + HER2‐: 62.3 HR + HER2+: 9	HER2: IHQ profiling and FISH test	HR + HER2− HR + HER2+ HR‐HER2+ and HR‐HER2‐	Age at diagnosis, histological grading, and survival rate
Ramírez Valle et al. [43]/Cuba (2019)	29.30	22.70	24.70	10.60	Luminal not specific: 12.6	NR	[40]	
González‐Longoria et al. [48]/Ecuador (2022)	53.80	11.60	14.60	20.10	—	NR	NR	
Vela et al. [49]/Ecuador (2020)	25.00	58.00	7.00	10.00	—	HER2: IHQ profiling and FISH test	[72]	
Ulloa et al. [50]/Ecuador (2020)	22.88	42.60	16.55	17.95	—	NR	[73]	Overall survival and disease‐free survival
Salazar‐Cifuentes et al. [57]/Guatemala (2017)	31.00	9.00	26.00	21.00	Luminal B‐like: 13	HER2: IHQ profiling and FISH test	[74]	Age at diagnosis, overall survival, and disease‐free survival
DeGennaro et al. [52]/Haiti (2018)			38.50	19.60	ER+: 51.8	HER2: IHQ profiling and FISH test [75]	NR	
Maffuz‐Aziz A., et al. [20]/Mexico (2017)			14.63	8.72	Luminal: 65.73 Luminal HER2 positive: 10.91	ER and PR positivity was defined as any positive nuclear staining in ≥ 1%–10% of tumor cells. HER2: IHQ profiling and FISH or CISH test	NR	
Reynoso‐Noverón et al. [21]/Mexico (2017)			16.00	23.20	HR positive and HER2 negative: 60.7	HR status was determined using the Allred score by IHC [76] HER2: IHQ profiling and FISH test [77]	HR positive, HER2 negative, HER2 positive, and triple‐negative	5 years OS
Reyna‐Sevilla et al. [22]/Mexico (2021)	31.60	27.80	15.00	8.80		NR	Luminal A: HR+, HER2−, luminal B: ER +/−, PR +/−, HER2 +, HER2/neu‐enhanced: HR−, HER2+, or triple‐negative	Age at diagnosis
Ornelas‐Aguirre et al. [23]/Mexico (2013)	33.72	6.71	28.52	18.12	Mixed (ER + PR + Her2Neu+): 12.91	HER2: IHQ profiling and FISH test	[78]	Stage at diagnosis, overall survival, and disease‐free survival
Pastén‐Zapata et al. [24]/Mexico (2019)	66.90	14.70	12.50	5.90		NR	NR	Stage at diagnosis and histological grading
Heredia‐Caballero et al. [26]/Mexico (2018)	35.80	27.30	19.60	16.30		NR	NR	
Valle‐Solís et al. [25]/Mexico (2019)			7.60	5.30	HR+, Her2‐: 69.6, HR+, and Her2+: 17.5	NR	HR+, Her2−, HR+, Her2+, Her2+, and HR‐Her2‐	Overall survival and disease‐free survival
Cabrera et al. [60]/Paraguay (2019)	36.00	25.33	21.33	9.33	Nonspecific: 8.01	NR	NR	
Medina Bueno [27]/Peru (2017)	37.50	31.40	14.60	16.40		ER and PR positivity were defined as any positive nuclear staining in ≥ 1% of tumor cells [79] HER2: IHQ profiling: Her2 (+++) [70]	2011 St. Gallen Consensus [68]	Age at diagnosis, axillary nodes involvement, histological grading, histological type, and overall survival
Garcés et al. [28]/Peru (2012)	58.2	10.10	21.60	10.10	RE+ 65.8; RP 52.3; Her2 20.2	ER and PR positivity were defined as any positive nuclear staining in ≥ 10% of tumor cells. For HER2, a result of 3+ is considered positive	Luminal A: ER+ and/or PR+, Her2Neu‐. Luminal B: ER+ and/or PR+, Her2Neu+. HER2‐enriched: ER−, PR−, Her2Neu+. triple negative: ER−, PR−, and Her2Neu‐	Age and stage at diagnosis, axillary nodes involvement, tumor size, histological grading, lymph vascular invasion, overall survival, and disease‐free survival
Chachaima‐Mar et al. [29]/Peru (2020)	40.15	32.43	15.44	11.97	RE+: 69.50; RP+: 59.07; Her2: 25.87	ER and PR positivity were defined as any positive nuclear staining in ≥ 1% of tumor cells [80] HER2:IHQ profiling and CISH test were performed on tumors with a score of 2+ [71]	2011 St Gallen Consensus [68]	Histological grading
Abad‐Licham et al. [30]/Peru (2018)	16.00 (St Gallen 2011) 28.00 (St Gallen 2013)	46 (St Gallen 2011) 34.00 (St Gallen 2013)	17 (St Gallen 2011) 17.00 (St Gallen 2013)	20 (St Gallen 2011) 20.00 (St Gallen 2013)		ER and PR positivity were defined as any positive nuclear staining in ≥ 10% of tumor cells HER2: IHQ profiling and for indeterminate cases, weak or incomplete staining (2+) CISH test was performed	2011/2013 St Gallen Consensus [66, 68]	Age at diagnosis
Galindo‐Céspedes et al. [31]/Peru (2016)	50.18	17.34	14.02	15.13		NR	Luminal A: ER/PR+, HER‐2/neu‐, luminal B (ER/PR+), Her2 neu+, nonluminal or Her2 (ER‐/PR− and HER‐2/neu+), and triple‐negative: ER‐/PR−, and HER‐2/neu‐ [81]	
Ortiz et al. [61]/Puerto Rico (2013)	61.8	3.3	17.3	7.5		RE and RP were reported as positive or negative. HER2: weak or incomplete staining (score +2) by IHQ profiling was excluded from the analysis	Luminal‐A ER and/or PR+, Her2−, Luminal‐B: ER and/or PR+, HER‐2+, and triple negative: HER‐2‐/ER‐/PR− HER‐2 overexpressing: HER‐2+, ER−, and PR‐	Age at diagnosis, histological type, and grading. overall survival
Chien et al. [39]/Venezuela (2012)	42.31	21.15	27.88	8.65		ER and PR positivity were defined as any positive nuclear staining in ≥ 1% of tumor cells [82]. HER2: IHQ profiling and FISH test	[83, 84, 85]	Stage at diagnosis, histological grading, overall survival, and disease‐free survival
Fernández‐Tortolero et al. [37]/Venezuela (2021)	72.7 (St Gallen 2009), 29.2 (St Gallen 2011), 26.8 (St Gallen 2013), 37.3 (St Gallen 2015)	27.3 (St Gallen 2009), 70.8 (St Gallen 2011), 73.2 (St Gallen 2013), 62.7 (St Gallen 2015)	NR	NR	Her2+: 12.9	ER and PR positivity were defined as any positive nuclear staining in ≥ 1% of tumor cells. HER2: IHQ profiling	St Gallen Consensus 2009 [86], 2011 [68], 2013 [66], and 2015 [87]	Overall survival
Uribe et al. [40]/Venezuela (2010)	60.63	1.56	28.75	9.06		Unclear	Luminal A: ER+ and PR+/−, HER2−, Luminal B: ER+ and PR+, HER2+; Basal‐like: ER−, PR−, and HER2−; and HER2+ enriched: ER−, PR−, and HER2+ negativos, and c‐erB‐2 positivo	NR
Bolívar Abreu et al. [38]/Venezuela (2013)			27.80	11.30	ER+: 60.9	NR	ER positive, HER2 enriched, and triple negative	Overall survival

Abbreviations: CISH: chromogenic in situ hybridization; ER: estrogen receptor; FISH: fluorescence in situ hybridization; HR: hormone receptor; IHC: immunohistochemical; LUM: luminal; NR: not reported; OS: overall survival; PR: progesterone receptor; SS: cancer‐specific survival; TN: triple negative.

Other studies have classified subtypes more broadly, defining luminal subtype as hormone receptor‐positive (HR+) and distinguishing between triple‐negative and HER2‐enriched subtypes based on IHC results (e.g., HER2+ when reported as 3+). HER2/enriched subtypes were reported in 34 studies. In 14 studies [9, 10, 15, 18, 20, 21, 23, 33, 35, 39, 49, 52, 55, and 57], HER2 assessment was conducted using IHC profiling combined with fluorescence in situ hybridization (FISH) or chromogenic in situ hybridization (CISH) (*n* = 3) [20, 29, 30].

The Luminal A subtype was the most frequent (36.95%), followed by Luminal B (28.72%), resulting in a combined total of 65.67% for all luminal subtypes. The most common classification method used was the St. Gallen panel, reported in nine studies across different years: 2009: one study [53], 2011: seven studies [10, 15, 27, 29, 30, 35, and 37], and 2013: four studies [9, 30, 33, and 37]. Applying the surrogate classification criteria proposed by the 2011 St. Gallen panel consensus, the Luminal B subtype was identified as the most prevalent (43.02%), followed by the Luminal A subtype (25.45%). According to the 2013 St. Gallen panel criteria, the prevalence of the Luminal B subtype was 40.26%, whereas the Luminal A subtype accounted for 24.6%. Specifically, using the surrogate classification criteria proposed by the 2011 and 2013 St. Gallen panel consensus, the Luminal B subtype was the most frequent in seven [9, 10, 15, 30, 33, 35, and 37] out of nine studies [9, 10, 15, 27, 29, 30, 33, 35, and 37], followed by Luminal A. These findings reflect a shift in the molecular subtype distribution and highlight the prevalence of Luminal B in studies from Brazil [9, 10, 15, and 18], Colombia [33, 35], Ecuador [49, 50], Peru [30], and Venezuela [37].

The triple‐negative subtype was reported in 33 studies, with an overall incidence of 17.45%. The highest prevalence was observed in Haiti (38.5%), whereas the lowest was found in Argentina (5.85%). The HER2‐enriched subtype accounted for 12.69% of all cases, with the highest occurrence observed in Bolivia (29.7%) and the lowest in Argentina (4.2%). There is a notable paucity of studies utilizing genomic platforms for subtype characterization. Only one study in this review included a basal‐like phenotype [33].

We observed differences in the distribution of intrinsic subtypes based on age (*n* = 17) [10, 15, 22, 27, 28, 30, 33, 46, 55, 57, and 61], histological grade (*n* = 10) [15, 24, 27, 28, 29, 33, 39, 46, 55, and 61], and tumor stage (*n* = 8) [9, 17, 23, 24, 28, 39, and 46]. Six studies reported a significant association between younger women and triple‐negative phenotypes [10, 15, 22, 30, 33, 46, 57], whereas four studies identified a similar association with HER2‐enriched phenotypes [28, 30, 46, 55]. Additionally, two Brazilian studies [10, 15] and one Peruvian study [44] observed a correlation between younger women and the Luminal B subtype [9, 16].

In nine studies, high‐grade tumors were significantly associated with the triple‐negative subtype and/or with the HER2 overexpressing subtype [15, 27, 28, 29, 33, 39, 46, 55, 61]. In four studies [9, 24, 39, 46], early stages were statistically significant for luminal subtypes.

### 3.3 Survival Outcomes

We included 28 studies that reported survival outcomes [10, 11, 12, 13, 14, 15, 16, 17, 18, 19, 21, 23, 25, 26, 27, 28, 32, 34, 38, 39, 41, 42, 45, 51, 52, 53, 55, and 61] (Table 4). In Latin America, there is a scarcity of studies evaluating BC survival beyond 5 years post‐diagnosis. Notably, only three studies from Brazil reported 10‐year survival data [12, 13, and 16]. For 5‐year survival, we analyzed 19 studies [9, 11, 14, 15, 16, 17, 18, 19, 21, 23, 26, 27, 28, 32, 38, 39, 42, 53, and 61] from eight countries: Brazil (*n* = 7) [10, 11, 12, 13, 14, 15, 16, 17, 18], Mexico (*n* = 4) [19, 21, 23, 26], Peru (*n* = 2) [27, 28], Venezuela (*n* = 2) [38, 39], Bolivia (*n* = 1) [53], Colombia (*n* = 1) [32], Cuba (*n* = 1) [42], and Puerto Rico (*n* = 1) [61]. For survival of less than 5 years, five studies were identified in Argentina (*n* = 1) [45], Colombia (*n* = 1) [34], Costa Rica (*n* = 1) [55], and Haiti (*n* = 2) [51, 52].

**TABLE 4:** Survival of selected studies of patients with breast cancer in Latin America.

Author/Year	Facility or facilities involved	Survival analysis type (start point and cause of death)	Survival rate	Study covariate
Abriata et al. [45]/Argentina (2019)	Multicentric: Institutional Cancer Registry of Argentina I (RITA)	Kaplan–Meier (unclear, NR)	OS at 12 months: 96%; and at 36 months: 84.7%	Age at diagnosis, *surrogate molecular subtype*, *lymph node involvement*, and *clinical stage at diagnosis*
Cruz‐Guisbert [53]/Bolivia (2019)	Single Center; Social Security University Hospital	Kaplan–Meier (NR)	OS at 104 months: 50%	
Peres et al. [10]/Brazil (2023)	Multicentric: São Paulo's hospital‐based cancer registry (RHC/SP) and the Immunohistochemistry Laboratory database at the Oncocenter Foundation of São Paulo (FOSP)	Kaplan–Meier (diagnostic, any cause)	OS at the 5th year: 72.5%	Age at diagnosis, education level, *histological type*, *clinical stage at diagnosis*, *tumor size*, lymph node involvement, time between diagnosis and treatment, type of treatment, hormone receptor status, and *surrogate molecular subtype*
Fujimoto et al. [11]/Brazil (2019)	Single Center: High Complexity Treatment Unit (UNACON) in Rio Branco, Acre State	Kaplan–Meier (diagnosis, cancer‐specific)	OS at the 1st year: 95.5%, 2nd year: 83.7%, and 5th year: 87.3%	Age at diagnosis, skin color, marriage status, tobacco smoking, alcohol consumption, comorbidity, second primary site cancer, family history of cancer, and the number of relatives with cancer. *Tumor size*, *clinical stage at diagnosis*, histological type, nodal status, and grade. *Estrogen receptor expression*, *progesterone receptor expression*, *HER2 Neu status*, and the type of treatment
Schneider et al. [14]/Brazil (2009)	Mutilcentric: Santa Catarina Center for Cancer Research and the Irmandade Nosso Senhor dos Passos Charity Hospital	Kaplan–Meier (diagnosis, cancer‐specific)	OS at 12th month: 95.7%, at the 24thmonth: 88.3%, at the 36th month: 83.4%, 48th month: 79.4%, and 60th month: 76.2%	*Age at diagnosis*, marriage status, race, *clinical stage at diagnosis*, histological type, and the type of treatment
Ayala et al. [12]/Brazil (2019)	Single Center: Unified Health System Mastology Service in Joinville, State of Santa Catarina	Kaplan–Meier (diagnosis and cancer‐specific)	OS at the 10 year: 41%	*Age at diagnosis*, family history, *clinical stage at diagnosis*, *histological grading lymph nodes involvement*, and metastasis
Balabram et al. [13]/Brazil (2013)	Single Center: Clinical Hospital of the Federal University of Minas Gerais, in Belo Horizonte, Minas Gerais State	Kaplan–Meier (treatment, cancer‐specific)	OS at the 5th year: 78.5% and OS at the 10th year: 64.5%	*Age at diagnosis*, *tumor size*, *regional lymph node status*, *clinical stage at diagnosis*, bilateral cancer, histological type, *histological grading*, and the type of treatment
de Macêdo Andrade et al. [15]/Brazil (2014)	Single Center: Fundação de Assistência da Paraíba” (FAP) public hospital of Campina Grande, Paraíba, Brazil	Kaplan–Meier (diagnostic, any cause)	NR	Histological type, grading, size, *surrogate molecular subtypes*, state of lymph nodes, and presence or absence of distant metastases. Age at diagnosis, menopause status, and patients' ancestry
Fayer et al. [16]/Brazil (2016)	Single center: One of the three High Complexity Care Units in Oncology of Juiz de Fora, state of Minas Gerais, Brazil	Kaplan–Meier (diagnosis, any cause)	OS at 10‐year survival: 56.3%	Age at diagnosis, skin color, *tumor size*, *lymph node involvement*, *clinical stage at diagnosis*, estrogen receptor, progesterone receptor, HER2, the type of health service, private health insurance coverage, time elapsed between diagnosis and surgery, and the type of treatment
Simon et al. [17]/Brazil (2019)	Multicentric: 28 Brazilian institutions	Kaplan–Meier (date of the surgery, any cause)	OS at the 5th year 88.74%	*Clinical stage at diagnosis*, histological type, *hormone receptors*, *HER‐2 status*, and *surrogate molecular subtypes*
Marques et al. [18]/Brazil (2022)	Single Center: Cancer facility in the state of Sergipe	Kaplan–Meier (unclear, any cause)	OS at 5 years: 50.5; 5‐year‐specific survival: 52.0	Age at diagnosis, *clinical stage at diagnosis*, histological type, *lymph node involvement*, and *surrogate molecular subtype*
Pardo et al. [34]/Colombia (2018)	Single center: Colombian National Cancer Institute	Kaplan–Meier (date of entry at the institute, any cause)	OS at the 2ndyear: 79.6	Age at diagnosis, *clinical stage at diagnosis*, Social Security Scheme, and years of entry at hospital
Zuluaga‐Liberato et al. [32]/Colombia (2016)	Single center: Oncological Center in Bogota	Kaplan–Meier (unclear, any cause)	OS at 5 years: 92.5	Age at diagnosis, *clinical stage at diagnosis*, histological grading, lymph node involvement, *hormone receptor*, and HER2Neu receptor status
Srur‐Rivero et al. [55]/Costa Rica (2014)	Single Center: San Juan de Dios Hospital	Kaplan–Meier (diagnosis, any‐cause)	OS at 45.6 months: 88	Age at diagnosis, *clinical stage at diagnosis*, histological grade, *surrogate molecular subtype*, *lymphovascular invasion*, *local recurrence*, and *distant recurrence*
Ricardo‐Ramírez et al. [41]/Cuba (2013)	Single Center: “Saturnino Lora Torres” Hospital in Santiago de Cuba	Kaplan–Meier (NR)	NR	*Age at diagnosis*, *clinical stage at diagnosis*, and histological type
García Soto et al. [42]/Cuba (2019)	Single center: “José Ramón López Tabranes” Hospital in Matanzas	Kaplan–Meier (diagnosis and cancer‐specific)	OS at 12 months: 94, OS at 25 months, OS at 77 months: 58, and OS at 5 years: 66	*Clinical stage at diagnosis*, *tumor size*, and *axillary lymph node involvement*
Fadelu et al. [51]/Haiti (2020)	Single Center: University Hospital Mirebalais	Kaplan–Meier (presentation date, any cause)	Event‐Free Survival rates—2 years: 80.9 and 3‐years: 63.4%	*Clinical stage at diagnosis*, histological grading, *estrogen receptor (ER) status*, treatments, and outcomes, including disease progression, and recurrence
DeGennaro et al. [52]/Haiti (2018)	Multicentric: Hospital Bernard Mevs and St Luke's Hospital in Port‐au‐Prince	Kaplan–Meier (first consultation, any cause)	OS at 12‐months: 81.6	*Clinical stage at diagnosis*
Álvarez‐Bañuelos et al. [19]/Mexico (2016)	Single Center: State Cancer Center (CECAN) in Xalapa, Veracruz, Mexico	Kaplan–Meier (diagnosis, any cause)	OS at 5 years: 63	*Age at diagnosis*, pregnancy, abortion, menopause, comorbidities, distant metastases, treatment, histological grading, histological type, *tumor size*, *axillary lymph node involvement*, e*strogen receptor*, *progesterone receptor*, and *HER2 status*
Reynoso‐Noverón et al. [21]/Mexico (2017)	Single Center: National Cancer Institute, Mexico City	Kaplan–Meier (diagnosis, any cause)	OS at 5‐years: 82	*Age at diagnosis*, menopausal status, body mass index, reproductive risk factors, and comorbidities. *Clinical stage at diagnosis*, histological type, histological grading, and *hormone receptor (HR) and HER2 status*
Ornelas‐Aguirre et al. [23]/Mexico (2013)	Single Center: Mexican Social Security Institute, Northwest National Medical Center, Sonora	Kaplan–Meier (NR, NR)		*Clinical stage at diagnosis*, histological grade, lymph node involved, and *surrogate molecular subtype*
Heredia‐Caballero et al. [26]/Mexico (2018)	Single Center: Women's Military Hospital, Mexico City	Kaplan–Meier (diagnosis, any cause)	OS at 5‐years: 81	*Clinical stage at diagnosis*, histologic grading, type, tumor size, axillary lymph node involvement, and molecular subtypes
Valle‐Solís et al. [25]/Mexico (2019)	Single Center: National Medical Center, November 20th, Social Security Institute, Mexico City	Kaplan–Meier (diagnosis, any cause)	NR	Age and clinical stage at diagnosis, tumor size, axillary lymph node involvement, *surrogate molecular subtype*, and treatment
Garcés et al. [28]/Peru (2012)	Single Center: National Institute of Neoplastic Diseases, Lima	Kaplan–Meier (surgery, any cause)	OS at 5‐years: 86	*Surrogate molecular subtypes*
Medina Bueno [27]/Peru (2017)	Single Center: Carlos Alberto Seguín Escobedo National Hospital	Kaplan–Meier (surgery, any cause)	NR	Histological type and s*urrogate* m*olecular subtype*
Ortiz et al. [61]/Puerto Rico (2013)	Mutilcentric: I. Gonzalez Martınez Oncological Hospital and the Auxilio Mutuo Hospital	Kaplan–Meier (NR, any cause)	OS at 5 years: 71.2	*Age and clinical stage at diagnosis*, surrogate *molecular subtype*, histological type, histological grading
Chien et al. [39]/Venezuela (2012)	Single center: Oncological Institute “Dr. Miguel Pérez Carreño,” Valencia	Kaplan–Meier (diagnosis, cancer specific)	NR	Age and c*linical stage at diagnosis*, *surrogate molecular subtype*, and *histological grade*
Bolívar Abreu et al. [38]/Venezuela (2013)	Single Center: Oncological Institute “Dr. Luis Razetti,” Caracas	Kaplan–Meier (diagnosis, any cause)	OS at 5 years: 74.4	*Surrogate molecular subtypes*

*Note:* Study covariate statistically significance were highlighted in italic.

Abbreviations: NR: not reported; OS: overall survival.

As anticipated, survival outcomes varied widely across studies. Findings from these observational cohorts in various countries indicate that 5‐year survival rates range from 50.5% [18] to 92.5% [32]. The overall 5‐year survival rates reported are as follows: Colombia: 92.5% (*n* = 1) [32], Peru: 86% (*n* = 1) [28], Mexico: 75.3% (*n* = 3) [19, 21, 26], Venezuela: 74.4% (*n* = 1) [38], Puerto Rico: 71.2% (*n* = 1) [61], Brazil: 75.62% (*n* = 6) [10, 11, 13, 14, 17, 18], and Cuba: 66% (*n* = 1) [42].

Across these studies, the independent prognostic factors associated with poorer survival outcomes included: advanced stage at diagnosis (*n* = 21) [10, 11, 12, 13, 14, 16, 17, 18, 21, 23, 26, 32, 34, 39, 41, 42, 45, 51, 52, 55, and 61], large tumor size (*n* = 6) [10, 11, 13, 16, 19, and 42], positive lymph node status (*n* = 8) [10, 12, 13, 16, 18, 19, 42, and 45], higher histologic grade (*n* = 3) [12, 13, 39], and HER2‐enriched and triple‐negative molecular subtypes (*n* = 18) [10, 11, 15, 17, 18, 19, 21, 23, 25, 27, 28, 32, 38, 39, 45, 51, 55, and 61]. The role of age as a prognostic factor was examined in 18 studies [10, 11, 12, 13, 14, 15, 16, 18, 19, 21, 25, 32, 34, 39, 41, 45, 55, and 61]. Poorer survival rates were reported for younger women (*n* = 3) [14, 19, 61] and older adults (*n* = 5) [10, 12, 13, 21, 41]. However, in 10 studies [11, 15, 16, 18, 25, 32, 34, 39, 45, and 55], age was not found to be statistically significant in predicting survival outcomes. Although regional differences between public and private care have been previously identified [88], this study found no survival disparities by health coverage status or social security scheme in the cohorts that reported these data [16, 17, and 34].

## 4 Discussion

This scoping review identified 54 primary studies across 19 countries in Latin America [9, 10, 11, 12, 13, 14, 15, 16, 17, 18, 19, 20, 21, 22, 23, 24, 25, 26, 27, 28, 29, 30, 31, 32, 33, 34, 35, 36, 37, 38, 39, 40, 41, 42, 43, 44, 45, 46, 47, 48, 49, 50, 51, 52, 53, 54, 55, 56, 57, 58, 59, 60, 61, 62]. These cohorts reveal significant clinical‐pathological heterogeneity within the region. This diversity can be attributed to the variable ancestry and genomic backgrounds across populations [2], which contribute to the distinct biological behavior of BC in Latin America.

One of the most notable features of our study population—consistent with cancer registries from LATAM and U.S. Hispanic/Latina populations—is the higher incidence of BC diagnoses before age 60 years, with fewer cases at older ages [89, 90]. In this review, the mean age was 54.28 years ± 2.75 years. This age distribution likely reflects the younger population structure, as well as the influence of socioeconomic, genetic, and lifestyle‐related risk factors [90].

In this study, advanced breast cancer (stages III and IV) accounted for 36.13% of cases, aligning with the 41% reported in a meta‐analysis of Latin America and the Caribbean [6]. This high proportion is due to low disease awareness—evident in Hispanic women in the U.S., who have a 1.19‐fold increased likelihood of presenting with later‐stage BC compared with non‐Hispanic White women [91]—and the limited access to healthcare in Latin America. Ongoing disparities in health coverage contribute to fragmented health systems, which provide minimal care, limited to urgent needs [4]. In contrast, Brazil, Cuba, and Costa Rica report universal health coverage. Additionally, Costa Rica, Chile, Colombia, Mexico, and Brazil have implemented universal healthcare models with expanded oncology services aimed at reducing catastrophic health expenditures and improving early detection. Whereas several of these countries report coverage rates above 90%, others maintain coverage at 45% or lower [92].

Genomic profiling offers essential prognostic and predictive insights into breast cancer but remains largely inaccessible in these countries due to high costs and operational complexity. As a result, intrinsic subtypes are typically inferred using IHC‐based surrogates. However, the lack of standardization across laboratories—driven by variable antibody selection, reagent inconsistency, and dearth of centralized quality control—undermines the reproducibility of IHC results. Furthermore, confirmation of equivocal HER2 status via in situ hybridization is limited by regional availability [4].

These limitations were reflected in our literature review, where heterogeneity in surrogate molecular phenotype classification emerged due to inconsistent terminology and methodologies across studies. To address this, we reported the full extent of variability, highlighting the urgent need for standardized protocols and quality control measures in breast cancer diagnostics throughout Latin America.

The elevated prevalence of Luminal B tumors identified in our review may reflect underlying genetic admixture and geographic commonalities among the included Latin American populations. However, differences in laboratory assessment of ER, PR, and Ki‐67 expression preclude definitive conclusions and do not exclude the potential for a similarly high prevalence of Luminal B in other countries.

Among Hispanic/Latina women in the U.S., the Luminal A subtype predominates (44.2%), followed by Luminal B (24.0%), HER2‐enriched (15.6%), and triple‐negative/basal‐like (11.6%) [7]. In contrast, our findings showed a slightly lower frequency of Luminal A (36.95%), with higher proportions of Luminal B (28.72%), triple‐negative (17.45%), and HER2‐enriched (12.69%) subtypes. Data from China report a similar pattern, with Luminal A at 41.1%, Luminal B 34.1%, HER2‐enriched 13.8%, and triple‐negative at 11.0% [93]. In Africa, the pooled frequencies of luminal, HER2‐positive, and TNBC subtypes are estimated at 56.3%, 12.6%, and 28.1%, respectively [94]. Our observed TNBC prevalence is consistent with other reports [95]. Despite the interstudy heterogeneity, our analysis confirms that TNBC and HER2‐enriched subtypes are consistently associated with younger age at diagnosis, higher histologic grade, advanced stage, and poorer prognosis.

These observational cohort studies report 5‐year breast cancer survival rates ranging from 50.5% to 92.5%, with an average of 76.41%, based primarily on data from local and regional hospital registries. Population‐based cancer registries cover only 6% of the Latin America population, in stark contrast to 96% in the U.S. and 32% in Europe [4]. The latest CONCORD study [96] using population‐based data for breast cancer revealed a reported 4‐year survival rate of 71.1% in Cali (Colombia), compared with 86.7% in Costa Rica and just 39% in Cuenca (Ecuador), reflecting the highest and lowest estimates in the region, respectively. In countries with optimal health system performance, BC survival exceeds 85%. The average 5‐year breast cancer survival rate in Latin America slightly exceeds 70% [97]. Long‐term survival data beyond 5 years are limited and predominantly derived from Brazilian cohorts [12, 13, 16], which report a 10‐year survival rate of 53.93%. In contrast to the US and Western Europe, where survival rates are approximately 20% higher [4, 6], these findings highlight persistent disparities in early detection and access to care.

Comparisons between countries should be interpreted with caution due to heterogeneity across studies. Differences in study periods, populations, data quality, statistical methods, and biases related to screening access, inclusion criteria, and patient selection may impact reported outcomes. Selection bias is likely in studies lacking consecutive patient inclusion, whereas missing data from retrospective sources further compromise analytical robustness. Outcome variability is also influenced by disparities in stage at diagnosis, age distribution, tumor biology, cancer surveillance systems, socioeconomic conditions, population structure, and healthcare infrastructure. These factors likely contribute to the observed discrepancies, particularly when compared with international, population‐based registries that use relative survival estimates.

This study has several limitations. Some cohorts were diagnosed before the routine implementation of key adjuvant therapies, such as anthracyclines, taxanes, and trastuzumab. Missing data from retrospective chart reviews and nonstandardized pathology reporting further limit the interpretability of findings. Advancing regional research requires targeted national initiatives and the removal of systemic barriers to data equity. Uniformed data collection is essential to characterize breast cancer heterogeneity and inform personalized care models to improve outcomes for Latina patients.

In conclusion, this scoping review underscores the clinical and pathological heterogeneity of breast cancer in Latin America, shaped by complex interactions between genetic ancestry, socioeconomic inequities, and healthcare system limitations. Despite the heterogeneity in diagnostic standards and reporting practices, consistent regional patterns in tumor subtypes, staging, and survival outcomes emerge. These findings highlight the urgent need for context‐adapted cancer control strategies and the development of personalized therapeutic approaches tailored to the region's unique demographic and molecular landscape.

## Author Contributions

**María Eugenia Aponte‐Rueda:** conceptualization, formal analysis, investigation, methodology, project administration, resources, supervision, validation, visualization, writing – original draft, writing – review and editing. **Fela Mar Gómez‐González:** data curation, investigation, resources, visualization. **Belén Merck:** conceptualization, formal analysis, investigation, methodology, project administration, resources, supervision, validation, visualization, writing – original draft, writing – review and editing.

## Conflicts of Interest

The authors declare no conflicts of interest.

## Supporting information


Supporting Information S1
